# Relation between migraine pattern and white matter hyperintensities in brain magnetic resonance imaging

**DOI:** 10.1186/s41983-018-0027-x

**Published:** 2018-09-10

**Authors:** Mohamed Negm, Ahmed Mohamed Housseini, Mohamed Abdelfatah, Alshimaa Asran

**Affiliations:** 1 Neurology, Faculty of Medicine, Suez Canal University, Ismailia, Egypt; 2 Diagnostic Radiology, Faculty of Medicine, Suez Canal University, Ismailia, Egypt; 3 Neuropsychiatry, Faculty of Medicine, Suez Canal University, Ismailia, Egypt

**Keywords:** Migraine, Aura, White matter hyperintensities (WMHs), MIGSEV scale

## Abstract

**Background:**

Migraine is a common disorder in general population. Presence of white matter hyperintensities (WMHs) in brain MRI of migraine patients was not studied clearly. Detection of the prevalence of white matter hyperintensities in migraine patients determines its correlation with migraine severity, type and duration.

**Methods:**

Cross sectional analytic study was conducted on migraine patients attending neurology clinic Suez Canal University Hospital. Sixty-five patients with migraine aged from 18 to 50 years were included. We excluded smokers and patients with hypertension, cardiac disease, diabetes mellitus, endocrine dysfunction, oncological and hematological diseases, infectious diseases, demyelinating disorders, and Alzheimer disease. Brain MRI and laboratory investigation was done for all patients.

**Results:**

White matter hyperintensities were significant more frequent in migraine with aura than those without aura. According to MIGSEV scale, white matter hyperintensities were highly significantly more frequent in grade III severity than grades II and I. The number of white matter hyperintensities increases significantly with increase intensity of pain during attack. The number of white matter hyperintensities increases significantly with increase intensity of nausea, disability, tolerability during attack and age. Resistance to treatment also shows statistically significant difference in increase number of WMHs.

**Conclusions:**

White matter hyperintensities are present in 43.1% of migraine patients. Age, presence of aura, nausea, disability during attack, resistance to treatment, and severity of headache and duration of migraine are considered a risk factor for development of white matter hyperintensities.

## Background

Headache disorders are among the most common disorders of the nervous system (World Health Organization 2017). Migraine is the third most common disease globally affecting 14.7% general population worldwide (Steiner et al. 2013). In Egypt, migraine prevalence was 10.51% among different types of headache (Kandil et al. 2016). According to the Burden of Disease Study 2013, migraine was the sixth highest cause of disability worldwide, measured in years of life lost to disability (YLDs) (Global Burden of Disease Study 2013 Collaborators 2015).

After imaging era, migraine is not thought to be a benign disease as it was before. Kruit et al. (2004a) consider migraine to be an independent risk factor for structural brain changes in magnetic resonance imaging (MRI) like deep white matter hyperintensities, silent posterior circulation territory infarcts, and infratentorial hyperintense lesions.

White matter hyperintensities (WMH) are referred to lesions that appeared in MRI that has infarction features but did not cause any clinical symptoms or other stroke-related signs. These lesions have common distribution in subcortical and deep white matter. In migraine with aura patients, WMH were found in deep white matter in 47% and in the periventricular region in 19% in contrast to small vessel disease where WMH found mainly at periventricular region (Masuda et al. 2001). These lesions found to be clinically important as WMH found to be associated with increasing cognitive impairment and increasing the risk of dementia by twofolds, while increasing risk for stoke by threefolds (Debette and Markus 2010). Also, WMH increase the risk of late onset depression (Herrmann et al. 2007), physical disability (Sachdev et al. 2005), and Parkinson’s disease (Beyer et al. 2006).

The aim of this work was to identify the prevalence of white matter hyperintensities among migraine patients and to determine the correlation between white matter hyperintensities and migraine severity, type and duration.

## Methods

The study was carried out as cross-sectional analytic study. We included 65 migraine patients aged from 18 to 50 years old attending the neurology clinic, Suez Canal University Hospital, and fulfilling the International Headache Society (IHS) criteria of migraine (Cephalalgia 2013). We excluded smokers and patients with hypertension, dyslipidemia, diabetes mellitus, hyperthyroidism, cardiac disease, history suggestive of any malignancy, and family history of Alzheimer disease. We excluded also patients with history suggestive of vascular, demyelinating, infection or other disorders with CNS pathology and patients with focal neurologic deficits and psychiatric illness.

Complete medical history done for all selected subjects. Assessment of migraine severity had done by using *MIGSEV scale* (El Hasnaoui et al. 2004) as follow:

A. Intensity of pain: (1) mild, (2) moderate, (3) intense, (4) very intense
B. Duration of attack, h: (1) < 4, (2) 4–12, (3) 12–24, (4) > 24
C. Nausea: (1) non, (2) mild, (3) intense, (4) vomiting
D. Disability: (1) no, (2) mild, (3) marked, (4) confined to bed
E. Tolerability: (1) tolerable, (2) barely tolerable, (3) intolerable
F. Resistance to treatment: (1) no, (2) yes
G. Frequency of attacks: (1) < 4/year, (2) 5–10/year, (3) 1–2/month, (4) 1/week, (5) > 1/week

Severity of migraine defined according to the main four component factors of the MIGSEV scale (Intensity of pain, Disability, Tolerability and Nausea) as: low (grade1) if at least 1 of the 4 items with a minimum score, and no item with a maximum score; high (grade 3) if at least 1 of the 4 items with a maximum score, and no item with a minimum score OR at least 2 items with a maximum score; and intermediate (grade 2) is all other cases.

Full clinical examination including neurological evaluation was done for detection of patients with focal neurological deficit. The following laboratory investigations were done for all the patients: lipid profile, fasting blood glucose, and thyroid-stimulating hormone levels.

### Imaging protocol

Brain MRI was done for all patients. All MRI studies were carried out at Radiology Department, Suez Canal University Hospital, using 1.5 Tesla MR Imager (Achieva; Philips Medical Systems, Best, Netherlands). Circularly polarized clinical head coils were used in all patients. Sequences done are T1-weighted spin-echo sequence (Repetition Time “TR”/Echo Time “TE”: 450/15), T2-weighted turbo spin-echo sequence (TR/TE: 2455/110; echo train length “ETL”: eight), FLAIR (Fluid Attenuation Inversion Recovery) sequence (TR/TE: 6000/120; inversion time “TI”: 2000) and diffusion weighted (DW) images with the b value = 1000 s/mm^2^. The matrix was 256 × 256, and the slice thickness was 5 mm, with a gap of 1 mm for all pulse sequences. For standard and accurate axial slice positioning, the anterior and posterior commissural line (AC-PC line) was used as a reference for T2-weighted and FLAIR images.

### Image analysis

All original DICOM images were transferred to Philips workstation for image analysis by the same radiology consultant (AM Housseini, with 16 years of experience as a general radiologist) who was blinded to the clinical data of patients. White matter hyperintensities, either unilateral or bilateral, are detected as high-signal-intensity punctate foci on T2WI and FLAIR images most commonly in the white matter of the centrum semiovale, contrary to small high-signal-intensity lesions seen at deep white matter of ischemic brain changes. Patients were divided into two groups: those with white matter hyperintense punctate foci and those without any lesions. WMH were considered if visible as hyperintense on T2-weighted and FLAIR images, without hypointensity on T1-weighted scans, and were larger than 3 mm.

### Statistical analysis

Data were fed to the computer and analyzed using IBM SPSS software package version 20.0 (Armonk, NY: IBM Corp) (Kirkpatrick and Feeney 2013). Qualitative data were described using number and percent. The Kolmogorov-Smirnov test was used to verify the normality of distribution. Quantitative data were described using range (minimum and maximum), mean, standard deviation, and median. *p* value is considered as significant when *p* < 0.05 and highly significant when *p* < 0.01.

The used tests were Mann Whitney test (for abnormally distributed quantitative variables, to compare between two studied groups); Kruskal Wallis test (For abnormally distributed quantitative variables, to compare between more than two studied groups, and Post Hoc “Dunn’s multiple comparisons test” for pairwise comparisons); and Spearman coefficient (to correlate between two distributed abnormally quantitative variables).

### Ethical approval and consent to participation

The study protocol was approved by the ethics committee of the Faculty of Medicine of Suez Canal University 20 July, 2016, registration number is 2877.

The purpose of the study was explained, and an informed written consent was taken before taking any data or doing any investigations. The participants were informed that their participation was voluntary and that they could withdraw from the study at any time without consequences. The participants were assured that all data will be considered confidential and will not be used outside this research. At the end of this study, records will be destroyed appropriately.

## Results

This study included 65 patients with migraine. Their age ranged from 18 to 50 years with mean age 28.98 ± 10.16 years. Forty-eight patients (73.8%) were females, and 17 patients (26.2%) were males.

We found that 55 patients (84.6%) of the patients had migraine without aura and 10 patients (15.4%) had migraine with aura; 8 of them has visual aura and 2 has sensory aura. According to severity of migraine, 32 patients (49.2%) were grade I, 20 patients (30.8%) were grade II, and 13 patients (20.0%) were grade III. Thirty-three patients (50.8%) of the studied patients had migraine for less than 5 years, and 18 (27.7%) patients had migraine for 5–10 years, while 10 patients (15.4%) had migraine for 10–15 years, and 4 patients (6.2%) had migraine for more than 15 years.

According to MIGSEV scale: intensity of pain showed that 22 patients (33.8%) had mild pain, 27 patients (41.5%) had moderate pain, 6 patients (9.2%) had intense pain, and 10 patients (15.4%) had very intense pain. According to duration of headache attack, 17 patients (26.2%) had attacks less than 4 h, 27 patients (41.5%) had attacks last for 4–12 h, 11 patients (16.9%) had attacks last for 12–24 h, and 10 patients (15.4%) had attacks last for more than 24 h (Table 1).

**Table 1:** MIGSEV scale in studied cases (*n* = 65)

	No.	%
Pain intensity during attack		
Mild	22	33.8
Moderate	27	41.5
Intense	6	9.2
Very intense	10	15.4
Duration of headache attack		
< 4 h	17	26.2
4–12 h	27	41.5
12–24 h	11	16.9
> 24 h	10	15.4
Frequency of attacks		
< 4 attacks/year	2	3.1
5–10 attacks/year	7	10.8
1–2 attacks/month	23	35.4
1 attack/week	18	27.7
> 1 attack/week	15	23.1

According to nausea scale, 33 patients (50.8%) had no nausea, 21 patients (32.3%) had mild nausea, 7 patients (10.8%) had intense nausea, and 4 patients (6.2%) had vomiting. According to disability during attack, 31 patients (47.7%) had no disability, 22 patients (33.8%) had mild disability, 8 patients (12.3%) had marked disability, and 4 patients (6.2%) became confined to bed. According to tolerability of headache during attack, 44 patients (67.7%) had tolerable headache, 16 patients (24.6%) had barely tolerable headache, and 5 patients (7.7%) had intolerable headache. According to resistance to treatment, 54 patients (83.1%) did not have resistance, while 11 patients (16.9%) had resistance (Table 2).

**Table 2:** MIGSEV scale in studied cases (continued) (*n* = 65)

	No.	%
Nausea		
None	33	50.8
Mild	21	32.3
Intense	7	10.8
Vomiting	4	6.2
Disability during attack		
No	31	47.7
Mild	22	33.8
Marked	8	12.3
Confined to bed	4	6.2
Tolerability during attack		
Tolerable	44	67.7
Barely tolerable	16	24.6
Intolerable	5	7.7
Resistance to treatment		
No	54	83.1
Yes	11	16.9

**Fig. 1:**
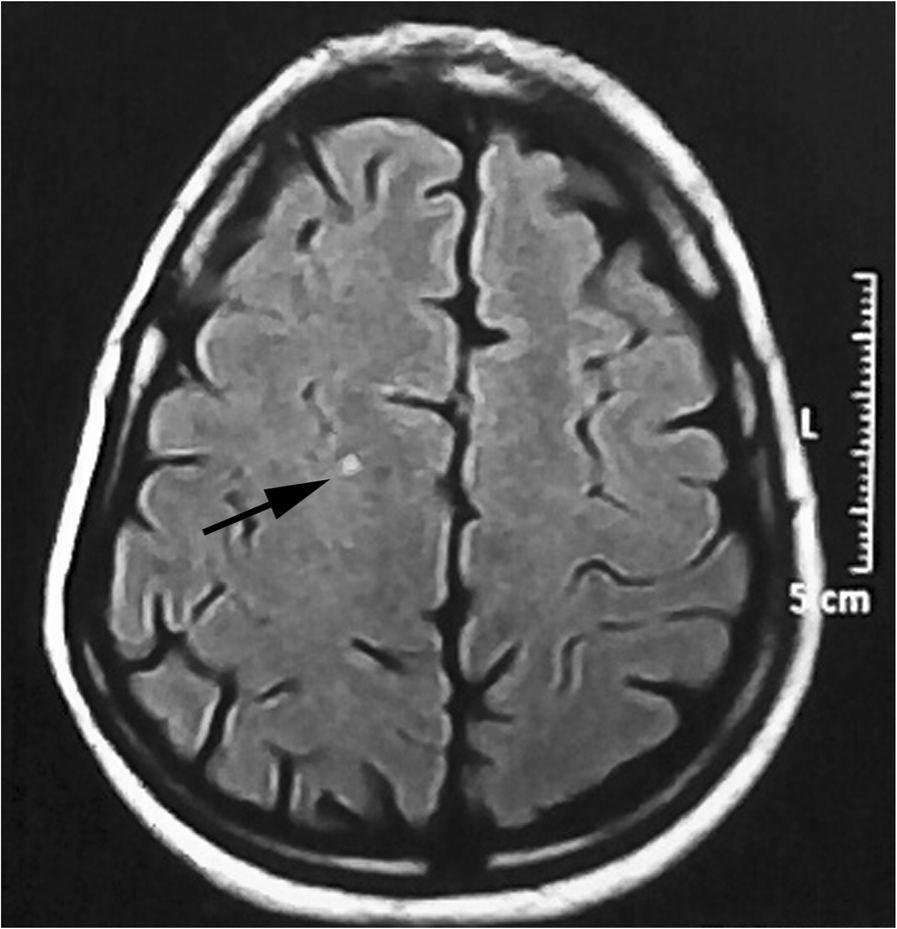
50-year-old female patient, not known to have any chronic illness, presented with migraine with aura for 10-year duration of grade III severity. Axial FLAIR MRI image shows small single bright focus at the right centrum semiovale (arrow)

**Fig. 2:**
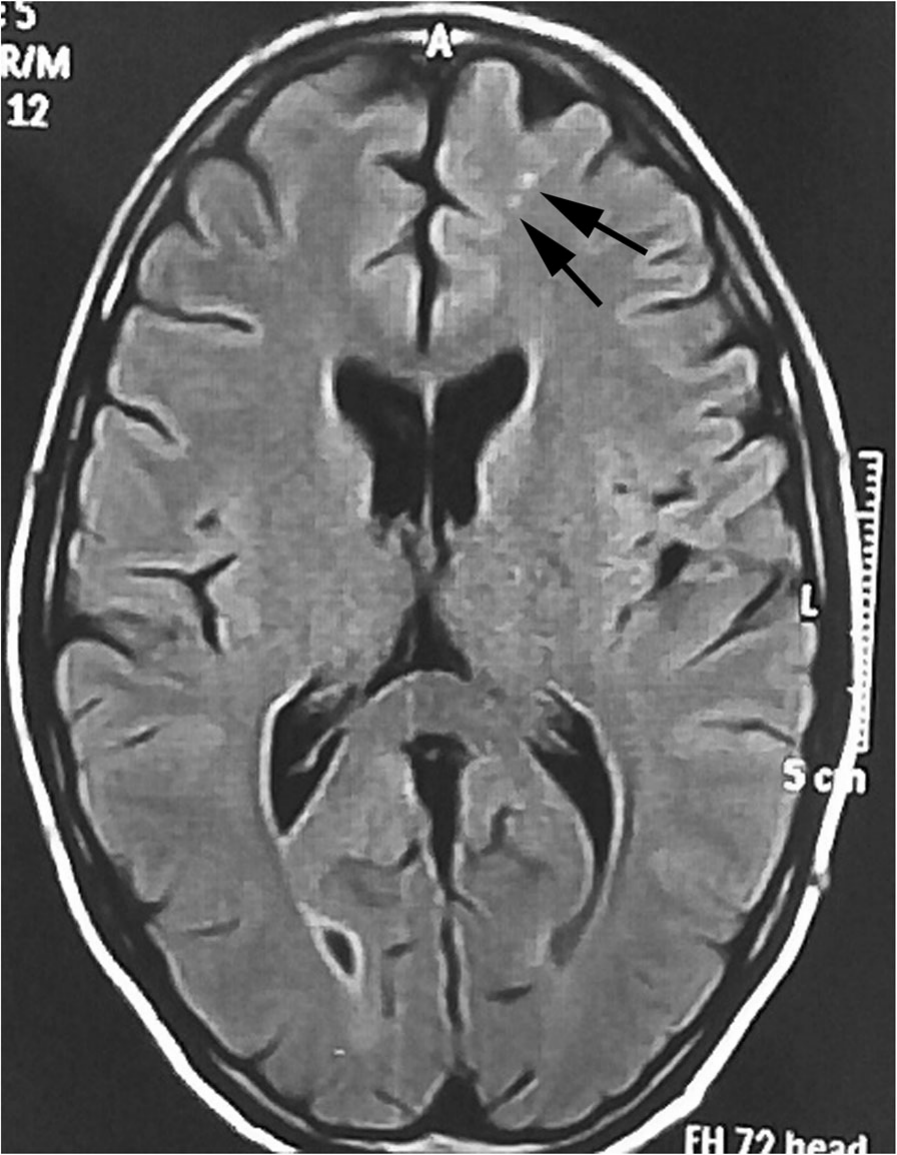
25-year-old female patient, not known to have any chronic illness, presented with migraine without aura for 6-year duration of grade II severity, not responding to medical treatment. Axial FLAIR MRI image shows two left frontal white matter hyperintense lesions (arrows)

According to number of white matter hyperintensities in MRI, 28 patients had white matter hyperintensities (43.1%), six of them had one lesion (9.2%) (Fig. 1), nine patients had 2 lesions (13.8%) (Fig. 2), and the rest 13 patients had more than 2 lesions (20.0%) with the highest number is 9 lesions with mean 1.22 ± 1.83 lesions.

There was no statistical significant difference detected between white matter hyperintensities as regard to sex**.** White matter hyperintensities were statistically significant more frequent in migraine with aura with median (2.90 ± 2.60) with maximum number of hyperintensities 9 lesions, while in migraine without aura the median was (0.91 ± 1.49) with maximum number of 6 lesions**.** There was no statistical significant difference detected between type of aura and white matter hyperintensities**.** According to grade of migraine, white matter hyperintensities were highly statistically significant more frequent in grade III severity with median (2.62 ± 2.69) with maximum number of hyperintensities 9 lesions, while grade II severity has median (1.55 ± 1.73) and maximum number of hyperintensities 6 lesions, and finally grade I severity has median (0.44 ± 0.88) and maximum number of hyperintensities 3 lesions**.** There was no statistical significant difference detected between duration of migraine and white matter hyperintensities (Table 3).

**Table 3:** Relation between number of white matter hyperintensities in MRI and sex, migraine type, severity, duration and type of aura

	*N*	Number of WMH in MRI			Test of sig.	*p*
		Min.–Max.	Mean ± SD	Median		
Sex						
Male	17	0.0–9.0	1.24 ± 2.28	0.0	*U* = 390.0	0.765
Female	48	0.0–6.0	1.21 ± 1.68	0.0		
Type of migraine						
Migraine with aura	10	0.0–9.0	2.90 ± 2.60	3.0	*U* = 127.0	0.003*
Migraine without aura	55	0.0–6.0	0.91 ± 1.49	0.0		
Type of aura						
Visual	8	0.0–9.0	3.50 ± 2.56	3.0	*U* = 1.50	0.084
Sensory	2	0.0–1.0	0.50 ± 0.71	0.50		
Migraine severity grade						
Grade I	32	0.0–3.0	0.44 ± 0.88	0.0	*H* = 13.703*	0.001*
Grade II	20	0.0–5.0	1.55 ± 1.73	1.0		
Grade III	13	0.0–9.0	2.62 ± 2.69	2.0		
Migraine duration						
0–5 years	33	0.0–9.0	0.82 ± 1.78	0.0	*H* = 5.482	0.140
5–10 years	18	0.0–5.0	1.44 ± 1.65	1.0		
10–15 years	10	0.0–5.0	1.90 ± 1.85	2.0		
More than 15 years	4	0.0–6.0	1.75 ± 2.87	0.50		

*U*, *p*—*U* and *p* values for Mann Whitney test. *H*, *p*—*H* and *p* values for Kruskal Wallis test. *WMH* white matter hyperintensities

*Statistically significant at *p* ≤ 0.05

The number of white matter hyperintensities found to increase significantly with increase intensity of pain during attack as mean number of hyperintensities in very intense pain (3.0 ± 2.91), intense pain (2.0 ± 1.26), moderate pain (0.93 ± 1.57), and mild pain (0.55 ± 0.96). While duration of attack and frequency of attacks show no statistical significant difference in number of white matter hyperintensities (Table 4).

**Table 4:** Number of white matter hyperintensities and MIGSEV parameters

	*N*	Number of WMH in MRI			*H*	*p*
		Min.–Max.	Mean ± SD	Median		
Pain intensity during attack						
Mild	22	0.0–3.0	0.55 ± 0.96	0.0	13.594*	0.004*
Moderate	27	0.0–5.0	0.93 ± 1.57	0.0		
Intense	6	1.0–4.0	2.0 ± 1.26	1.50		
Very intense	10	0.0–9.0	3.0 ± 2.91	2.50		
Duration of attack						
< 4 h	17	0.0–3.0	0.76 ± 1.20	0.0	3.815	0.282
4–12 h	27	0.0–5.0	0.93 ± 1.33	0.0		
12–24 h	11	0.0–6.0	1.55 ± 2.25	0.0		
> 24 h	10	0.0–9.0	2.40 ± 2.88	1.50		
Frequency of attacks						
< 4 year	2	0.0–3.0	1.50 ± 2.12	1.50	6.316	0.177
5–10 years	7	0.0–6.0	2.14 ± 2.48	2.0		
1–2 month	23	0.0–4.0	0.57 ± 1.16	0.0		
1 week	18	0.0–9.0	1.50 ± 2.38	0.50		
> 1 week	15	0.0–4.0	1.40 ± 1.45	1.0		

*H*, *p*—*H* and *p* values for Kruskal Wallis test. *WMH* white matter hyperintensities

*Statistically significant at *p* ≤ 0.05

The number of white matter hyperintensities was found to increase significantly with increase intensity of nausea during attack as mean number of hyperintensities in patients who had vomiting (2.75 ± 2.50), intense nausea (2.29 ± 1.89), mild nausea (1.38 ± 1.66), and those did not have nausea (0.70 ± 1.70). Also disability and tolerability during attack show statistically significant difference in increase number of white matter hyperintensities. Mean number of hyperintensities to those patients confined to bed (2.0 ± 2.83), marked disability (2.38 ± 1.77), mild disability (1.82 ± 2.28), and those had no disability was 0.39 ± 0.76. While mean number of hyperintensities who had intolerable headache was 3.0 ± 2.24, barely tolerable 2.25 ± 2.54, and those has tolerable headache 0.64 ± 1.08. At the last, resistance to treatment also shows statistically significant difference in increase number of white matter hyperintensities as mean number of hyperintensities in patients who had resistance to treatment was 2.27 ± 1.74 and those who did not have resistance to treatment was 1.0 ± 1.79 (Table 5).

**Table 5:** Number of WMH and MIGSEV parameters (continued)

	*N*	Number of WMH in MRI			Test of sig.	*p*
		Min.–Max.	Mean ± SD	Median		
Nausea						
None	23	0.0–9.0	0.70 ± 1.70	0.0	*H* = 10.590*	0.014*
Mild	21	0.0–5.0	1.38 ± 1.66	1.0		
Intense	7	0.0–5.0	2.29 ± 1.89	2.0		
Vomiting	4	0.0–6.0	2.75 ± 2.50	2.50		
Disability during attack						
No	31	0.0–2.0	0.39 ± 0.76	0.0	*H* = 14.054*	0.003*
Mild	22	0.0–9.0	1.82 ± 2.28	1.0		
Marked	8	0.0–5.0	2.38 ± 1.77	2.50		
Confined to bed	4	0.0–6.0	2.0 ± 2.83	1.0		
Tolerability during attack						
Tolerable	44	0.0–4.0	0.64 ± 1.08	0.0	*H* = 11.860*	0.003*
Barely tolerable	16	0.0–9.0	2.25 ± 2.54	2.0		
Intolerable	5	0.0–6.0	3.0 ± 2.24	3.0		
Resistance to treatment						
No	54	0.0–9.0	1.0 ± 1.79	0.0	*U* = 151.5*	0.005*
Yes	11	0.0–5.0	2.27 ± 1.74	3.0		

*H*, *p—H* and *p* values for Kruskal Wallis test. *U*, *p*—*U* and *p* values for Mann Whitney test. *WMH* white matter hyperintensities

*Statistically significant at *p* ≤ 0.05.

According to multivariate analysis**,** neither gender nor duration of attack had statistically difference, but age, migraine severity grade, pain intensity during attack, nausea, disability, and tolerability had a highly statistically significant difference, while migraine duration and resistance to treatment had a statistically significant difference (Table 6).

**Table 6:** Multivariate analysis of development of WMH among migraineurs

	Number of WMH in MRI	
	*r*_s_	*p*
Age (years)	0.419*	0.001*
Males	− 0.037	0.768
Migraine severity grade	0.462*	< 0.001*
Migraine duration	0.276*	0.026*
Attack duration	0.211	0.092
Pain intensity	0.404*	0.001*
Nausea	0.402*	0.001*
Disability	0.444*	< 0.001*
Tolerability	0.428*	< 0.001*
Resistance to treatment	0.353*	0.004*

*r*_s_ Spearman coefficient. *WMH* white matter hyperintensities

*Statistically significant at *p* ≤ 0.05

## Discussion

In our study, we found that the prevalence of WMHs in migraine patients is 43.1% with different numbers of hyperintensities. This was consistent with Le Pira et al. 2013a who estimated the prevalence to be 43.2%. Similarly, Seneviratne et al. 2013a also estimated the prevalence to be 43%. A higher prevalence was reported by Rossato et al. 2010 who estimated it to be 56.4% among migraineurs with aura; their high frequency might be due to studying patients who had migraine with aura only. Whereas a lower prevalence was noted in other studies (Zeytin et al. 2014 (11.53%), Toghae et al. 2015 (32.2%), Zhang et al. 2016 (32%), and Trauninger et al. 2011 (31.18%)), the low incidence in these studies may be because of different methodology.

Our study showed that there is no statistical significance as regard to sex and the presence of WMHs. This finding was supported by other studies (Toghae et al. 2015; Zhang et al. 2016; Trauninger et al. 2011; Gomez-Beldarrain et al. 2016; Chong and Schwedt 2015; Hamedani et al. 2013; Kruit et al. 2004b).

In our study, presence of WMHs was higher in migraine with aura than migraine without aura. This comes in accordance with Le Pira et al. 2013b, Bashir et al. 2013, and Kruit et al. 2010. This could be explained by fluctuations in cerebral blood flow associated with hyperperfusion or hypoperfusion which modulated by cortical spreading depression from recurrent aura attacks that affect micro-vascular hemodynamics leading to ischemic injury (Rothwell 2010). In the opposite side, Zhang et al. 2016 and Hamedani et al. 2013 showed that migraine without aura had more WMHs load than those with migraine with aura. While other studies (Toghae et al. 2015; Trauninger et al. 2011; Seneviratne et al. 2013b) show that there was no difference between migraine subtype and WMHs, which may be due to differences in patient selection and sample size.

In our study, the high grade of severity of migraine was associated with more WMHs. Our finding was supported by Toghae et al. 2015, and this could be explained by the hemodynamic changes, neuronal activation or neurogenic inflammation, and disruption of blood brain barrier due to cortical spreading depression occurred in recurrent severe attacks of migraine (Seneviratne et al. 2013b). But Le Pira et al. 2013b did not find statistically significant relation between severity of migraine and WMHs; this may be small sample size.

There was no significant relation between the presence of WMHs and duration of migraine by years. This finding was supported by most of the other previous studies (Zhang et al. 2016; Le Pira et al. 2013b; Bashir et al. 2013; Seneviratne et al. 2013b; Szabó et al. 2012). On the other hand, Toghae et al. 2015 found a significant correlation between duration of migraine and WMHs. Also, Trauninger et al. 2011 concluded that patients with migraine disease more than 20 years had WMHs more frequent than patients with migraine disease less than 20 years. That contradiction might be because in our study most of migraineurs had migraine less than 10 years. Only 4 patients had migraine for more than 15 years.

In our study, increased intensity of pain of headache during attack had a significant relation to increased WMHs incidence. Toghae et al. 2015 did not find the same relation; use of different scales to assess the intensity of pain may be the explanation. On other hand, both attack duration and frequency did not have significant relation to develop WMHs in our study which might suggest that these changes are specific to migraine and not only to the chronic, repeated painful condition. These results were supported by other studies (Rossato et al. 2010; Toghae et al. 2015; Zhang et al. 2016; Szabó et al. 2012). On other hand, Seneviratne et al. 2013b and Trauninger et al. 2011 considered attack frequency is associated to WMHs, explained by tissue damage during attacks caused by local inflammatory responses and excessive neuronal activation during attacks. This contradiction in results may be due to differences in patient selection and sample size.

In our study, disability, tolerability during attacks of headache, and intensity of nausea were found to have high statistical significance in increasing number of WMHs. Also resistance to medical treatment as a sign of severity of migraine was strongly related to WMHs development. On other hand, Kruit et al. 2010 and Toghae et al. 2015 found no significant relation between the use of prophylactic drug use and WMHs.

Age was detected in our study to be a risk factor to develop WMHs in migraineurs. This might be because of increasing age means increase number of years lived with migraine which leads to more WMHs. Most of previous studies support our results (Rossato et al. 2010; Toghae et al. 2015; Zhang et al. 2016; Le Pira et al. 2013b; Seneviratne et al. 2013b). Meanwhile, Gomez-Beldarrain et al. 2016, Bashir et al. 2013, and Trauninger et al. 2011 show no relation between age and WMHs in migraineurs, which might be because of migraine itself decrease in frequency and severity with increasing age which means it remit with age.

Using Spearman’s correlation coefficient, we found that there is highly statistically positive correlation between WMHs and migraine severity grade according to MIGSEV scale, disability during attack and tolerability during attack making them the most important risk factors in developing WMHs that should to be controlled. While age, migraine duration, intensity of pain, nausea and resistance to treatment were found to be statistically correlated to WMHs, sex and attack duration were not statistically correlated.

The major strength points of our study is its clinical-based design, the standardized diagnosis of migraine according to International headache society criteria, and the sensitive MRI protocol that was read by an experienced neuroradiologist blinded to clinical data minimizing the possibility that lesions were misclassified. Limitations of the study included small sample size because of the strict selection criteria. Also the use of 3-T MRI could better visualize smaller lesions.

## Conclusions

In conclusion, migraine is commonly associated with WMHs (43.1%). Age, presence of aura, nausea, and disability during attack, resistance to treatment, and severity of headache and duration of migraine are considered a risk factor for development of WMHs, although WMHs were detected more frequent in grade III severity of migraine that is considered an association.

## Abbreviations

IHS: International Headache Society
WMHs: White matter hyperintensities
